# Exploring the experiences of resident doctors in child and adolescent psychiatry with virtual reality-based simulation training: a qualitative study

**DOI:** 10.1186/s12913-024-11941-w

**Published:** 2024-11-21

**Authors:** Siv Lena Birkheim, Giovanna Calogiuri, Mette Hvalstad, Randi Martinsen

**Affiliations:** 1 Department of Health and Nursing Sciences, Faculty of Social and Health Sciences, Inland Norway University of Applied Sciences, P.B. 400, 2418 Elverum, Norway; 2 Innlandet Hospital Trust, P.B 104, 2380 Brumunddal, Norway; 3 University of South-Eastern Norway, P.B 4, 3045 Drammen, Norway

**Keywords:** Focus groups, Lifelong learning, Observational learning, Reflective thematic analysis, VR-based learning, Virtual reality-based learning

## Abstract

**Background:**

The use of virtual reality simulation for the training of non-technical skills among healthcare professionals may improve professional development as well as the quality of care. This study aims to explore the experiences of resident doctors in child and adolescent psychiatry with the use of virtual reality-based simulation for the training of non-technical skills.

**Methods:**

The study had an exploratory qualitative design. Data were collected through observations of thirteen resident doctors in child and adolescent psychiatry during their participation in three virtual reality-based simulation sessions, followed by two focus groups. Data were analyzed using reflective thematic analysis.

**Results:**

The analysis revealed the following three themes: 1) The importance of pedagogical principles, 2) Playful and motionally affected learning, and 3) Observational learning contributing to lifelong learning.

**Conclusions:**

Virtual reality-based simulation was felt to be an appropriate method of training non-technical skills for resident doctors in child and adolescent psychiatry. In particular, the intervention met resident doctors’ desires and needs regarding learning through observation and peer discussions. However, some challenges related to technical issues and the content of the scenarios were highlighted. This method may not only increase the resident doctors’ self-efficacy and competence, but also contribute to their lifelong learning.

## Background

In recent years, there has been a global increase in mental ill-health among children and adolescents [1–3], including patients with multiple diagnoses requiring complicate treatment [4]. The workforce in mental healthcare must be adequately prepared to tackle this social challenge [5]. In this context, the training of non-technical skills (NTS; i.e., “the cognitive, social, and personal resource skills that complement technical skills, and contribute to safe and efficient task performance” [6] is paramount. However, guidelines and tools for evaluating [6] and training NTS in mental healthcare are missing [7, 8]. During residency or postgraduate training, which sees resident doctors combining education and work on the field, effective guidance and support are crucial [9]. However, poor standards of the learning processes during this formational period are a recurrent issue [9–11]. Among other aspects, residents and novice doctors often report being unprepared to keep up to date with the most recent standards of care, as well as having little time and opportunities to practice NTS such as communication and decision making [12, 13].

There is ever increasing adoption of technology in healthcare, with the field of health technology developing exponentially [14, 15]. However, it is debated to what extent different types of technology improve learning [16]. The medical education system is often slow to update its teaching methodology to new needs and requirements imposed by the field [17]. For instance, while healthcare simulation, digital learning, virtual reality (VR) and augmented reality (AR) have opened new doors in the treatment and management of patients, there is still limited knowledge of how these tools and techniques best support education as well as lifelong learning. VR-based learning can be seen as a playful way to learn, as the participants` imagination and intellect unite and become a tool to discover knowledge in their own way and pace [18]. Moreover, VR has been evaluated as improving visual memory [19], providing realistic environment, and giving a feeling of involvement- and a sense of excitement and joy [20]. In light of this, the inclusion of VR in the methodology of medical education curricula has attracted increasing interest, although research and implementation are still at an early stage [21].

VR is a broad concept with different tools and applications. One pathway through which VR can engage learners is the sense of presence that it can generate, which refers to the users subjective feeling and sense of actually being in the virtual environment [22]. Immersive VR refers to VR technology that presents a visual environment that convincingly replaces the user’s real-world surroundings [22], hence increasing the likelihood that the person will experience high sense of presence. An example of immersive an VR device which has gained popularity in recent years is the head-mounted display (HMD). The popularity of HMDs has been rapidly increasing in the past decade, largely thanks to the introduction on the market of consumer-oriented devices. Such technology has the advantage of being (relatively) inexpensive and user-friendly, allowing people with little technical expertise to access and enjoy a wide variety of VR experiences. Consumer-oriented cameras that allow users to create VR content are also available. The contents created through such cameras (also known as “360° videos”) can realistically replicate real environments or events. Unlike interactive computer-generated scenarios, 360°videos do not normally enable users to interact with virtual world (e.g., by “moving” in the virtual space or by influencing the virtual environment), as they are merely exposed to a pre-determined sequence of images.

Despite these limitations, 360° videos can have valuable applications in context where the aim is to simulate a real-life situation. In particular, 360° videos can reproduce cases in which actors simulate patient-therapist interactions, which can be used in the context of VR-based simulation learning.

### Virtual Reality based Simulation Innlandet (VR-SIMI)

VR-based Simulation Innlandet (VR-SIMI) is a program developed at the Centre for Simulation and Innovation of Innlandet Hospital Trust in Norway with the purpose of providing health care professionals with training in NTS [20]. The protocol, which is delivered in the form of group-based training sessions, consists of five stages: 1) clarification of objectives, 2) prebriefing, 3) visualizing a case through VR, 4) debriefing, and 5) application to practice. The recommended group size is five to eight participants. The cases are developed as 360° videos (VR scenarios) by experienced clinicians and cover a variety of clinical situations. Each VR-SIMI session, led by a trained facilitator, lasts about an hour.

In an observational field study, Birkheim et al., [20] explored the experience of healthcare professionals in child and adolescent mental healthcare who participated in a VR-SIMI training program. Through this study, an evidence-based and theory-driven pedagogical framework was proposed to guide the practical implementation of the training program. The framework highlights how VR-SIMI has an overall constructivist approach, where participants construct their own knowledge through a facilitated process. Specifically, VR-SIMI builds on Kolb’s experiential learning theory, which involves a continuous cycle of experience, reflection, conceptualization and active experimentation [23]. Overall, the learning process in VR-SIMI is framed within an adult learning context with specific learning objectives related to practice. Hence, VR-SIMI also aligns with the concept of situated learning [24], which emphasizes that learning occurs in a socio-cultural context. Further, VR-SIMI builds on social cognition theory and observational learning [25–27], especially with respect to observational learning, modelling and self-efficacy [28, 29].

VR in medical education has thus far been mostly used for individual training in technical skills, such as surgical procedures [12, 21, 30, 31], not for group training [21]. It is also used to teach non-technical skills such as empathy and communication by using virtual patients and avatars [32, 33]. The use of 360° videos as a tool in simulation-training of NTS in medical education, is still in its early stages [18, 30]. The application of the tool to training in NTS mental healthcare, including child and adolescent mental health, is still largely unexplored [34, 35]. The framework proposed by Birkheim et al. [20] is based on observations and spontaneous interviews with a heterogeneous group of healthcare professionals (psychologists, nurses, social environment workers, social workers and psychiatrists) in child and adolescents mental health services, who participated in VR-SIMI as part of their regular training in NTS. Other groups of healthcare professionals are likely to present different professional experiences and learning needs, which may influence their learning experience during VR-SIMI.

### Aim of the study

In order to further strengthen the proposed pedagogical framework, as well as to explore how the dynamics and procedures of VR-SIMI can be applied to other groups of healthcare professionals**,** the aim of the present study is to explore the experiences of resident doctors in child and adolescent psychiatry participating in a VR-SIMI as part of their residency training.

The following research question was developed to guide the study:

How is VR-SIMI experienced by resident doctors in child and adolescents’ psychiatry as a method to achieve learning outcomes in NTS during their residency training?

## Methods

### Setting of the study

This study is a part of the overarching project “Mental Health Care for Children and Youth” (https://nbup.no/bup/prosjektet/), led by the South-Eastern Norway Regional Health Authority. The overall goal of the project is to recruit and retain experienced staff and increase competence and development in mental healthcare for children and young people.

The participants attended three VR-SIMI sessions in an education centre where they commonly met for teaching and guidance. The participants view three scenarios with learning objectives adapted to the residency programme: 1) a clinical consultation with an aggressive adolescent related to compulsory treatment in mental healthcare, 2) an initial clinical assessment and interview with a child and its parents, 3) a confrontation with a colleague.

The VR-SIMI sessions lasted from one to one and a half hours. The study took place during the COVID-19 pandemic, which entailed a greater physical distance between participants, which would not be necessary under normal conditions. The VR-SIMI sessions were led by one trained facilitator and one trained supervisor, who cooperated throughout the session. This was a familiar arrangement during residency training.

### Design

The study had an exploratory qualitative design, including field observations and focus groups.

### Data collection and description of the participants

Data were collected during participant observations during three VR-SIMI sessions and two focus groups developed for this study, which were conducted shortly after the last session. Participant observation enables close observation of participants, allowing researcher to obtain detailed qualitative description of what the participants think, feel and do [36]. Because the observations provided limited time and opportunity to explore the topic in-depth, focus groups were conducted to explore common experiences and attitudes in the group [37]. Thirteen resident doctors (eleven women and two men) in child and adolescents psychiatry participated in at least one of the three VR-SIMI sessions over a period of three month. The participation rate was high, with the lowest number of participants in a session being eleven. One participant dropped out of the group before the focus group discussions for an unknown reason. Two participants were aged 20–30 years, nine were between 31–40 years old and two were in the age group 41–50 years. Eight of the participants had less than one year of experience in child and adolescent psychiatry, while five of them had from three to five years of experiences. Four participants had no previous experience with healthcare simulation as a teaching method, and only three had experienced VR before joining the VR-SIMI training programme. The participant observations were conducted by the first author during the VR-SIMI sessions and documented in a field diary.

The focus groups (n = 7 and n = 5) developed for this study, were conducted by the first author with the last author as a co-moderator. A thematic guide containing three themes guided the focus groups. In addition, the VR-SIMI manual was used in the focus groups to remind the participants of the theoretical and practical themes of the VR-SIMI method. The group discussions were recorded and transcribed verbatim.

### Data analysis

**Fig. 1:**
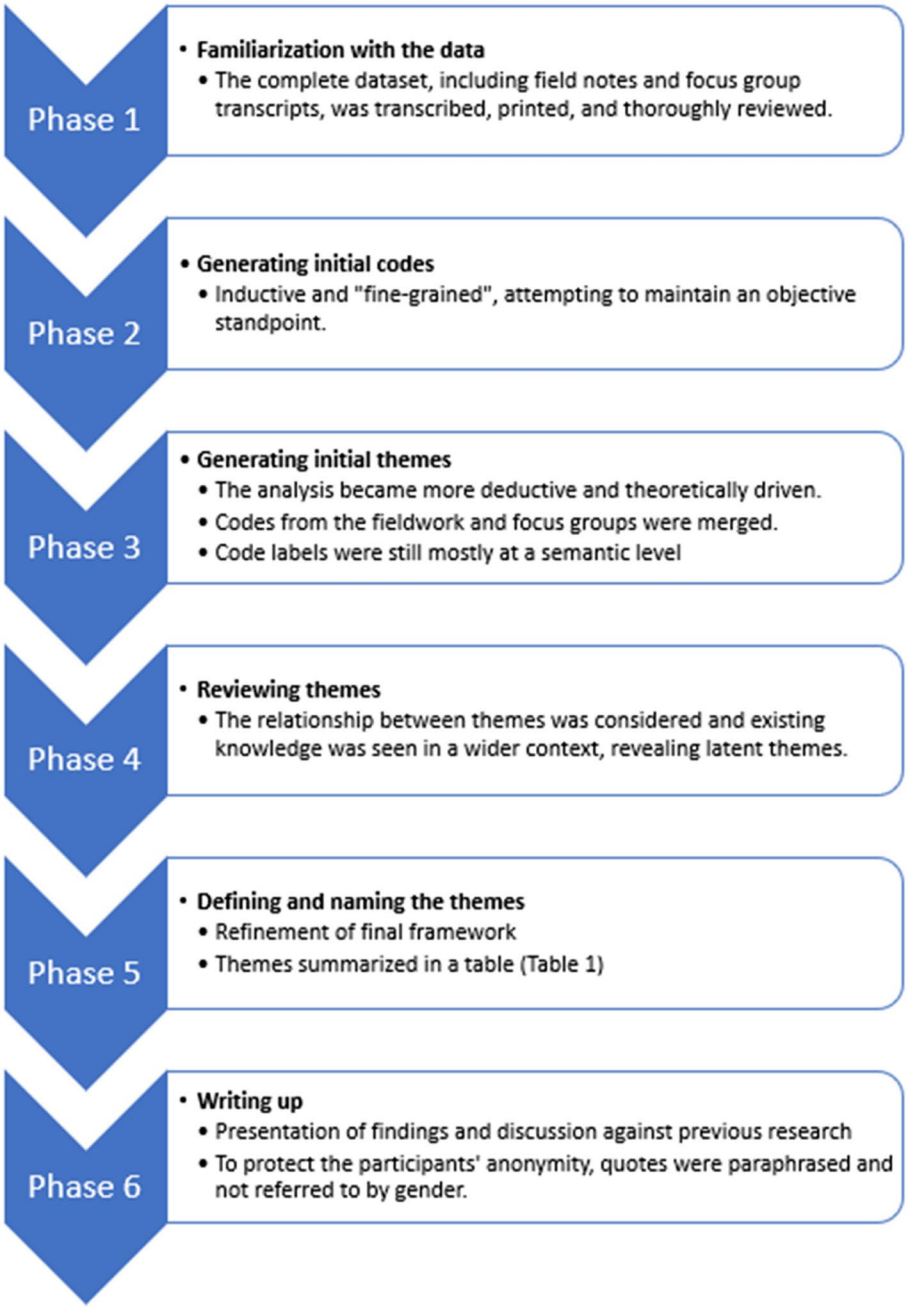
Flow diagram of the reflexive thematic analysis

Reflexive thematic analysis (RTA) was utilized to systematically identify, organize and provide insight into the patterns of meaning in the collected data. RTA is a suitable and beneficial analysis method for research on perceived experience and for explaining decisions made during the analysis process [38]. The analysis was approached in an abductive manner, which means that the themes were derived from both the data themselves and from the pedagogical framework of VR-SIMI [20] as a theoretical understanding of the topic. Braun and Clarke [39] suggest that RTA should focus primarily on semantic or latent themes, but in this analysis, it was useful to consider both in the different phases. Due to the small sample of participants, quotes were paraphrased to protect their privacy. Quotes are not referred to by gender, due to the skewed distribution. The six steps of the RTA process [39] is presented in Fig. 1, while the codes and themes (phase 2 to 5) are summarized in Table 1.

**Table 1:** Overview of the reflexive thematic analysis process

Phase 2: Coding Examples of codes:	Phase 3 and 4: Sematic and latent themes	Phase 5: Refining and naming themes (Latent themes)
-Scepticism -Easy technology -Fun - Learning objectives -Safety in the group	-Trying VR in a learning setting was amusing, exciting, refreshing, easy, effective, something new - Scepticism - The technology does not always work as planned - It was interesting, recognizable, the scenarios showed relevant topics - Clarifications of expectations is important - Relevant scenarios related to learning outcomes made it interesting - It is good that the facilitators use theory from the prebreif, their own experience and always link the reflection to the learning objectives - Good structure, one of the best classes	**The importance of pedagogical principles**
-Fun -Presence -Emotions -Effective way to learn -Professional mirror of emotions -Learning in all phases	- Like a fly on the wall - VR arouse strong feelings, you feel it in your body - The feeling of being in the room is the next best thing to sitting face to -face - Can feel the discomfort some intense conversations in practice might arouse - More involved personally in VR than other learning methods - Remember better with VR than other learning methods -VR-SIMI function as a professional mirror	**Playful and emotionally affected learning**
-Master apprentice learning -Recognizable scenarios -Learning of NTS -Practical	- Would like to have more scenarios and more VR-SIMI sessions - Learning occurs in prebreif, while watching the scenario in VR and in debrief - VR-SIMI is a good method to share your own knowledge and past experiences and to learn from others - VR-SIMI gives many potential solutions and no conclusions -VR-SIMI is safe, effective, and possible to combine in everyday work - VR-SIMI is preferred to medical simulation and roleplay	**Observational learning contributing to lifelong learning**

## Results

Based on the RTA, three main themes were identified: 1) The importance of pedagogical principles, 2) Playful and emotionally affected learning, and 3) Observational learning contributing to lifelong learning.

### The importance of pedagogical principles

The use of VR-SIMI as a new pedagogical method in the residency training program was primarily perceived as something new, exciting, interesting and fun. Initially, a few participants were sceptical as they had limited technical experience, and were not sure they could operate the HMD. However, this concern quickly disappeared when they realized that handling the HMD was both simple and self-explanatory. This was expressed during the first VR-SIMI session by one of the participants:

Technically, I’m surprised. The VR is so easy to use. That’s an important factor in considering if it will be used or not.

Nevertheless, some technical issues emerged, as the HMD “did not always work as planned”. Having quick access to a backup HMD and as a person with technical knowledge at hand was considered valuable, but not essential.

The size of the group (with up to 13 participants per session) was not seen as an obstacle to learning by the participants in the study, as stated by one of them:

As long as I feel safe in the group, size doesn't matter.

Indeed, a large group, including people with different ages, experiences and knowledge, was seen as a strength. One of the participants even stated that it might be a good idea to include other healthcare professionals, such as nurses, family therapists, psychologists or physiotherapists to achieve even deeper and broader reflections, who were felt to be lacking in the clinical practice. However, most of the participants stated that they would prefer conducting VR-SIMI in smaller groups. One of the participants explained:

You become more engaged when there are fewer people, because there are fewer people talking and there is no one to hide behind.

Smaller groups were felt to be less intimidating and facilitate more active participation in the reflection phase. Nevertheless, the most important factor was to have a pleasant, safe atmosphere within the group, to encourage all participants to share their thoughts and experiences. This came out clearly when one participant said:

It’s important that you don't have to be afraid of making a fool of yourself.

Several of the participants worked in small facilities in rural areas and complained of a lack of meeting places and opportunities to have discussions and share interesting topics with other professionals. Meeting colleagues and supervisors in VR-SIMI sessions was thought to be very valuable.

In view of the size of the group and the possibility to achieve the learning objectives, two of the main supervisors in the residency training program functioned as facilitators. The participants appreciated that the facilitators had considerable experience in the field, as well as being pleasant and non-judgmental. The fact that the facilitators contributed their own experience was perceived as a strength, especially in the debriefing phase.

This has been one of the best classes we have had in this program. Structured, efficient and with a lot of learning.

This is an example of showing that both pedagogical principles and facilitator qualities play a crucial role in enabling successful learning processes. The participants found it very useful that the facilitator was well- prepared and had a structured plan for the session.

The prebriefing was generally considered to be an important learning element in the VR-SIMI session.

I don’t think I would have been able to reflect in the same way if I hadn’t been prepared with theory in advance.

Prebriefing activities such as attending a lecture in advance and/or reading articles relevant to the learning objectives were thought to be useful by most participants.—However, their perceived need for a theoretical brief in advance varied greatly according to whether participants were novices or had several years of experience from the clinical field. It was also pointed out that the reading material should be handed out in advance to enable participants to find time to read it in their busy lives outside the residency program. Those who were unable to complete the prebriefing still had a learning. However, being better prepared could have enhanced their reflection in the group, as summarized by one of the participants:

The prebrief strengthens the learning process, as long as it is relevant to the learning objectives.

### Playful and emotionally affected learning

Quotes from the observation of participants emphasizes that watching the VR-scenario aroused their emotions. One who was a novice in using VR summarized this as followed:

You get something extra when you see the scenario in HMD compared with a TV screen, you get more, for example emotions. You feel the feelings of the people sitting in the room, while you also become aware of your own feelings.

Although the scenarios were somewhat emotionally charged, and the learning objectives serious, there was also a certain degree of playfulness, as highlighted by expressions such as:

This is fun, I get carried away.

This might be associated with the use of the novel, exiting technology, as well as the generally pleasant atmosphere in the room during the VR-SIMI sessions. The participants frequently smiled and laughed, and commented on the excitement of trying something new, being impressed by the technology, feeling calm and not being stressed about having to do roleplays/scenarios themselves as they do in medical simulation.

During the VR-SIMI sessions, the participants appeared deeply focused and concentrated on the topic and learning objectives, especially when they started viewing the VR scenario. Several stated that it was liberating to only have to concentrate on one task at the time and felt as if they were “disappearing” into the virtual world, indicating high levels of presence. This appeared to arouse and support the participants’ interest in the dynamics depicted in the VR scenario, which was also experienced as recognizable and realistic. This quote exemplifies how some participants felt carried away while viewing the VR scenario:

Having the opportunity to “be in the room “and feel real emotions is important for learning.

However, several of the participants noted that the scenarios were too short. The ten-minute scenarios made them curious about what would happen next. They felt that the scenarios were both realistic and useful to develop their professional role and found it pleasant and helpful to feel so close to recognizable situations. One participant explained:

I feel like a fly on the wall. This is the next best thing to being in the room myself.

Several participants commented that they would have preferred to see the same scenario a second time, after they had finished the collective reflection phase, as they got new thoughts and ideas after the reflection phase. More scenarios related to their residency training were called for.

### Observational learning contributing to lifelong learning

The sessions revealed that VR-SIMI might address a gap in the field of practice. One of the participants stated with a sigh:

Finally, we had the opportunity to see how those we want to learn from work themselves.

On the other hand, the participants also found that the scenarios were not fully in accordance with the realistic situation in the clinical field. For instance, some criticized the fact that all the scenarios included two therapists in the conversations, which was exceptional in their experience. Indeed, they reported often feeling alone during their clinical practice, which they found challenging, particularly the recent graduates who were in the early stages of their residency in child and adolescent psychiatry. In such cases, the capacity of the VR-SIMI training to foster self-efficacy may be limited, as the doctors may feel that they would be unable to handle a similar situation when *alone* with a patient.

In line with core principles of observational learning, being able to identify with a role model- (in this case, the therapists depicted in the VR scenario) is pivotal for learning to occur. Accordingly, the resident doctors found it particularly meaningful to recognize themselves in the “virtual” clinicians.

I think it’s great using VR-SIMI as one of the teaching methods in this residency program. Seeing others work means you learn something new and pick up some tricks. I’d like to experience many more scenarios.

The participants expressed concern that access to the VR-SIMI training would no longer be available after termination of the research project. They repeatedly praised VR-SIMI as a method to practice NTS as part of their everyday work, as they felt it was safe and effective.

Ideally, I would like to practice face-to-face, but this is as close as you can get to that feeling.

The participants had several suggestions for integrating VR-SIMI into their clinical practice and as part of their lifelong learning. Several also called “train- the trainer” course to become VR-SIMI facilitators themselves.

## Discussion

Negative influences during childhood and adolescence can catalyse behaviours that, in the long-term habits, may lead to adverse consequences and development of noncommunicable diseases [40]. Considering that mental ill-health can act as a risk factor for other chronic diseases [41], the global increase in mental ill-health among child and adolescents [1–3] is particularly concerning. Digital technology is increasingly becoming integral part of the prevention and management of noncommunicable diseases [42], including within mental health care [43]. The digitalization of health services involves also the education and training of healthcare professionals, such as for the case of VR-SIMI. However, the long-term effectiveness of digital learning tools is still largely unknown [44]. The aim of this study was to examine how VR-SIMI was experienced by resident doctors in child and adolescent psychiatry as a method to achieve learning outcomes in their residency training. The results provide further understanding of the application of the method and the learning processes that take place within VR-SIMI, expanding the evidence base supporting the framework previously proposed in Birkheim et al. [20].

The findings highlight how, as soon as the resident doctors put on the HMD, they became absorbed and engaged in the 360° scenarios. Established theories emphasize how visual aids can enhance learning outcomes- people learn better from pictures and words than words alone. For instance, Mayer’s cognitive theory of multimedia learning [45] emphasizes the importance and influence of the words and picture we select for educational purposes. Being able to observe a simulated familiar and interesting situation, relevant to clinical practice, created an engaging learning experience with an increased sense of presence among the participants in the study. This aligns with Slater and Wilburs [22] concepts of immersion and presence. In particular, wearing the HMD with headphones, which can be considered a highly immersive form of VR technology, was explicitly linked by the participants to high feelings of presence, and their subjective perception of being within the virtual experience [22]. The results further emphasize how such elevated feelings of presence elicited emotional awareness among the participants. They recognized feelings of anger and frustration similar to the feelings they experience in clinical practice. This is in line with previous studies investigating the way which emotional experiences are related to presence [46, 47]. This emotional impact is pedagogically valuable, as stronger emotional engagement is known to support learning [19, 48]. In particular, in accordance with theory on observational learning, for a modelling process to occur, various experiences must elicit emotional arousal [28, 29]. However, as highlighted by Makransky et al. [49], VR may overload learners and distract them away from the learning goal. This underlines the importance of basing VR-based training on sound and structured pedagogical principles and processes, rather than simply introducing a new technology with inadequate adjustments [20].

This study was conducted during the COVID-19 pandemic. Despite the challenges of restrictions such as social distancing and wearing masks, it was possible to conduct VR-SIMI training satisfactorily as planned. This shows that VR-SIMI is a reliable pedagogical method despite its various limitations [20]. Nevertheless, technical issues may occur- for instance as the HMD may malfunction, it is important to keep backup devices to allow for quick replacement with minimal impact on the training session. VR-SIMI provided scenarios where the resident doctors could explore their curiosities and professional knowledge and observe critically without running the risk of hurting the feelings of patients or the actors in the scenarios. VR technology provides engaging experiences in training and education as it can increase the frequency of authentic learning experiences by providing new arenas for active constructivist learning [17], which has been argued to be a primary goal in using VR in education [50]. This resonates with the ability provided by VR-SIMI to see the same scenario at different paces (e.g., pausing as needed), or by having different viewpoints of observations (e.g., by orienting one’s view at different angles in the 360° range of vision). The VR-SIMI structure, based on experiential learning theory, has an element of playfulness [18]. The fact that the participants found VR-SIMI an enjoyable experience provided greater motivation to use it and deeper commitment in the learning process, adding a new element to the learning. This aligns with studies on elementary school students, demonstrating that VR is evaluated as playful learning with increased motivation [51], and among final year medical students, where the immersive environment captured the students’ attention, as the sense of presence gave them a better understanding and a significantly increased learning experience [52]. The group context as well as manageable challenges (e.g., learning how to use the HMD), which support basic needs for relatedness and competence [53], also emerged as meaningful elements of the learning experience.

Individual emotions, motivation and engagement are all important aspects of learning [19], although often under-rated in professional training. Working with mentally ill children, youth and their relatives may be complex, unpredictable and lonely. Poor perceived competence and self-efficacy may add to the psychological workload, especially among young and inexperienced therapists, leading to burnout among residents [54]. Further, this emotional exhaustion combined with high work stress may reduce productivity, decrease the quality of patient care and may lead to high turnover [55], as a stressful workload and a lack of clinical supervision may increase anxiety and depression among residents [48]. As an association exists between commitment, motivation, and learning [17], VR-SIMI may represent an effective and attractive learning practice that can be integrated into the resident training (and beyond), enhancing residents’ competence in NTS, as well as their sense of security and self-efficacy in facing the challenges associated with their clinical practice.

Traditionally, the education of medical doctors was provided by more experienced doctors as role models and teachers, but with little attention to pedagogical principles [56]. This master-apprentice model worked to a certain extent, but schools lacked standardized requirements, and the link between theory, research and clinical practice was very limited [9]. Although outdated, the master-apprentice model has recognized merits. Having the opportunity to observe experienced healthcare professionals in action, as the resident doctors in this study did during the VR-SIMI sessions, was highlighted as a desired and needed element of their training program. Learning by observing the behaviour of others aligns with Bandura’s social learning theory [28] and relies on processes such as modelling and vicarious reinforcement. For these processes to occur, certain prerequisites need to be met, namely that the learner can relate to and identify with the observed model and that strong emotional responses are aroused. The findings of this study highlight how VR-SIMI supports such processes, strengthening the residents’ self-efficacy in adequately tackling similar situations in their practice.

### Positioning VR-SIMI within the context of healthcare training and clinical practice

In order to position VR-SIMI within healthcare training and clinical practice, it is pertinent to compare this method with more traditional healthcare simulation. In healthcare simulation, also known as medical simulation, some participants are required to participate actively through role-play, while others act as observers. While the active participation through role-play has beneficial educational value, the observer role may be given limited attention and focus on learning outputs [57]. By contrast, in VR-SIMI, the focus is primarily on the observer role. As confirmed through the findings of the present study, the HMD facilitate focused attention to emotional presence. This higher degree of involvement of the observers is crucial in the observational learning process, as explained by Bandura, during which the attention has to be directed toward the modelled behavior in order to learn from the model [28].

While the observational phase (i.e., when the participants view the scenario through HMD) is a pivotal feature of the VR-SIMI training, as in healthcare simulation, the other phases are essential to fully achieve the learning outcomes. From first spending time on the theory in the prebrief, to watching relevant scenarios, where the residents could observe and identify with the professionals played in the scenario, as a preparation to the clinical setting. Also vital was the discussion and sharing of feelings and thoughts in the reflection/debriefing phase, in a safe environment, without harming the patient or relatives. The reflection phase supports the search to find answer to how these feelings can be handled, which will in turn be important for resilience, learning and development. This aligns with what Goleman [58] points out as emotional intelligence.

Beyond the structured learning activities in the VR-SIMI sessions, the social context and networking value of the sessions also emerged as highly valuable for the learning experience, as well for the resident doctors’ professional growth. Finding space and time to reflect on or discuss various elements with colleagues or supervisors was mentioned by the participants in this study to be challenging, especially considering that many of them were deployed in different geographical locations, some in rural areas. Meeting in groups to attend the VR-SIMI sessions was commented on as a way to break the perceived loneliness in their work and having a synergistic effect on insights and solutions that go beyond the one-to-one relationship, as in individual guidance. This aligns with Lycke et al. [59], who highlights the benefits of supervision in groups, but also the obstacles to organizing groups. Professionals in medicine are persistently urged to engage in continuous professional development throughout their careers., lifelong learning [60, 61]. Hence, it is important to suggest tools that are effective, but also engaging. The findings of this study indicate that VR-SIMI, can address both these requirements, and may therefore be proposed as a useful tool in the lifelong learning of medical professionals. In particular, VR-SIMI emerged as capable of supporting basic psychological needs of autonomy, competence, and relatedness, which are paramount for supporting intrinsic motivation as well as psychological wellbeing in learners [62].

### Challenges and future direction

One of the challenges in adopting VR in learning setting is the supervisors/teachers lack of knowledge and experience of other learning methods than traditional methods, such as lectures in classrooms [17]. At the same time, there is relatively high motivation to use new technology such as VR [63], but a lack of anchoring in management, limited finance and low public willingness to invest are barriers to implementation. These barriers need to be investigated in further research. Further, even though VR-SIMI was generally positively received, some scepticism was mentioned, especially in the initial stages, before the participants tried the HMD. Although such scepticism was quickly overcome as soon as the participants could verify that the HMD was safe and easily manageable, this might highlight potential reluctance and barriers to participation. Finally, unrealistic scenarios (e.g., the predominance of case depicting two therapists working together) and a limited number of available scenarios for training call for further work on developing contents for VR-SIMI training that adequately addresses the needs of the trainees. In this respect, it may be advisable a ensure greater involvement of end-users in the development of such scenarios through co-creative or participatory processes.

### Strengths and limitations

This study addresses a novel and under researched pedagogical tool, thus expanding the evidence and knowledge relevant to VR-based simulation in general, as well as to the VR-SIMI method in particular. Observations during multiple VR-SIMI sessions, using both participant observation and focus groups, which were facilitated by two researchers, are methodological strengths of this qualitative study. The data were analyzed using an established analysis method, with the analysis process transparently presented, and the result further discussed and revised by fellow researchers.

However, a number of limitations need to be taken into considerations. Firstly, in this study, there was a relatively small sample of residents. With only thirteen participants included, the range of experiences, perspectives, and responses captured in through the focus groups might be limited [64]. This undermines the depth and generalizability of the findings. The fact that only three VR-SIMI sessions were observed limits the scope of the findings, especially in the extent to which they provide evidence of changes over time as well as achievement of planned learning outcomes in the long term (i.e., improving NTS). Moreover, at its current stage, the study cannot provide evidence on the viability of VR-SIMI as a resource for lifelong learning. Studies with longer observation periods are warranted to provide more detailed answer as to whether VR-SIMI is a suitable pedagogical method, especially in a life-long learning context. As another limitation, the generally positive evaluations of the participants, may have been inflated by an “expectation effect “or a “halo effect” [64],-i.e., the participants engagement in and their positive comments on the VR-SIMI sessions may have been influenced by the awareness that researchers were observing them.

## Conclusion

VR-SIMI was experienced as an appropriate method of training NTS among resident doctors in child and adolescents’ psychiatry. The findings indicate that the high levels of presence generated through the VR technology elicited- emotional awareness; and arousal, which are crucial for observational learning to occur. VR-SIMI seemed to meet the resident doctors wishes and needs with respect to observational learning as well as peer discussions, in order to provide security and self-efficacy in facing the challenges of being a resident doctor. Furthermore, VR-SIMI has the potential to be adapted to a wide range of learning objectives and to support lifelong learning. However, as the scientific evidence in this field is limited, further research is warranted, especially investigating the sustainability and effectiveness of this pedagogical methods in the long term, as well as its actual impact on competencies of resident doctors and other healthcare professionals.

## Data Availability

The transcripts (in Norwegian) are available from the corresponding author on reasonable request.

## Abbreviations

HMD: Head- mounted display
RTA: Reflexive thematic analysis
VR: Virtual reality
VR-SIMI: Virtual Reality-based Simulation Innlandet
NTS: Non-technical skills
